# The neurobiological synergy of transcranial magnetic stimulation and mindfulness-based interventions in treatment-resistant depression: a narrative review

**DOI:** 10.3389/fpsyt.2026.1805825

**Published:** 2026-04-02

**Authors:** Sonora Yun, Martijn Figee

**Affiliations:** The Department of Psychiatry, The Icahn School of Medicine at Mount Sinai, New York, NY, United States

**Keywords:** CEN, central executive network, default mode network, DMN, MBI, mindfulness based interventions, rTMS, salience network

## Abstract

Treatment-resistant depression (TRD) poses a significant challenge, with many patients failing to achieve remission with conventional therapies. While repetitive Transcranial Magnetic Stimulation (rTMS) is an established and effective neuromodulatory treatment for TRD, a notable proportion of patients do not fully respond. Despite the clinical utility of rTMS, response variability remains a significant barrier to treatment optimization. We propose that this variability may be driven, in part, by fluctuations in baseline network configuration and momentary brain states. This motivates the investigation of behavioral state manipulation as a means to prime neural circuits for enhanced receptivity to neuromodulation. This mini-review proposes that combining TMS with adjunctive, short-duration mental practices, specifically Mindfulness Based Interventions (MBI), may enhance therapeutic outcomes. Given that both TMS and mindfulness converge on modulating brain networks regulating emotions, attention and self-referential processes, it is plausible that their concurrent or sequential use could yield additive or even synergistic effects on network reorganization. We will explore the theoretical neurobiological mechanisms that may support a potential synergistic or additive effect and synthesize the limited but promising evidence from analogous therapeutic combinations to argue for the clinical viability of this novel approach. This review aims to: (1) Summarize the shared network targets (DMN, CEN, and SN) of rTMS and mindfulness; (2) Propose a state-dependent synergy model for their integration; and (3) Synthesize existing evidence to guide future clinical trial design.

## Introduction

Treatment-resistant depression (TRD) poses a significant challenge, with many patients failing to achieve remission with conventional therapies. While repetitive Transcranial Magnetic Stimulation (rTMS) is an established and effective neuromodulatory treatment for TRD, a notable proportion of patients do not fully respond. Despite the clinical utility of rTMS, response variability remains a significant barrier to treatment optimization. We propose that this variability may be driven, in part, by fluctuations in baseline network configuration and momentary brain states. This motivates the investigation of behavioral state manipulation as a means to prime neural circuits for enhanced receptivity to neuromodulation. This mini-review proposes that combining TMS with adjunctive, short-duration mental practices, specifically Mindfulness Based Interventions (MBI), may enhance therapeutic outcomes. Given that both TMS and mindfulness converge on modulating brain networks regulating emotions, attention and self-referential processes, it is plausible that their concurrent or sequential use could yield additive or even synergistic effects on network reorganization. To our knowledge, no review has yet synthesized the neurobiological rationale for integrating TMS with brief, structured mindfulness practice in TRD, despite evidence that both interventions engage overlapping neural substrates. We will explore the theoretical neurobiological mechanisms that may support a potential synergistic or additive effect and synthesize the limited but promising evidence from analogous therapeutic combinations to argue for the clinical viability of this novel approach.

In this review, TRD is defined as a failure to achieve remission after at least two adequate trials of antidepressant medications. Standard rTMS modalities discussed herein include high-frequency (HF-rTMS, 10 Hz) and intermittent theta-burst stimulation (iTBS) targeted to the left dorsolateral prefrontal cortex (dlPFC). The term “brief mindfulness primer” refers to a guided, 8–15 minute mental practice administered immediately prior to a stimulation session. While the general benefits of these therapies are known, few reviews have focused specifically on circuit-level mechanisms and state-dependence in pairing rTMS with brief mindfulness practice.

This review aims to: (1) Summarize the shared network targets (DMN, CEN, and SN) of rTMS and mindfulness; (2) Propose a state-dependent synergy model for their integration; and (3) Synthesize existing evidence to guide future clinical trial design.

## Methods

A literature search was conducted in PubMed/Medline and PsycINFO for articles published from inception through January 2026. The search strategy employed flexible combinations of terms across four primary domains: (1) neuromodulation (“TMS,“ “rTMS,“ “iTBS,“ “neuromodulation”); (2) behavioral interventions (“mindfulness, “ “meditation, “ “MBI”); (3) clinical targets (“depression,“ “TRD”); and (4) neurobiological markers (“default mode network,“ “DMN,“ “functional connectivity,“ “resting-state fMRI”). Inclusion criteria comprised of: (1) clinical studies combining rTMS with mindfulness or meditation, alongside analogous studies pairing rTMS with cognitive or behavioral tasks; or (2) clinical trials, meta-analyses, and mechanistic studies relevant to the state-dependent effects of mindfulness or rTMS on large-scale brain networks. Non-human studies and those not involving depression or analogous psychiatric populations were excluded. As a narrative mini-review, a formal risk-of-bias scoring was not performed, though selection was guided by the SANRA framework to ensure thematic coherence and reporting quality.

## Background

### The neurobiological basis of tms and mindfulness meditation

Mindfulness meditation is the deliberate mental practice of nonjudgmental, present-moment awareness, typically involving a focused orientation toward immediate sensory experiences—such as breath, posture, and affective states—coupled with a cognitive stance of openness and acceptance (1, 2). Consistent mental practice has been shown to induce measurable structural neuroplasticity, providing a robust neural substrate for enhanced self-regulation. Specifically, mindfulness training has been shown to increase gray matter density and white matter integrity within the anterior cingulate cortex (ACC) (3, 4). These structural changes may provide the anatomical basis for improved functional coupling between prefrontal control hubs and limbic structures, supporting a “triad” of regulatory mechanisms: enhanced attentional control, improved emotion regulation, and reduced self-referential processing (1, 2). On a network level, this physical reorganization manifests as enhanced functional integration among large-scale brain networks.

**Figure 1:**
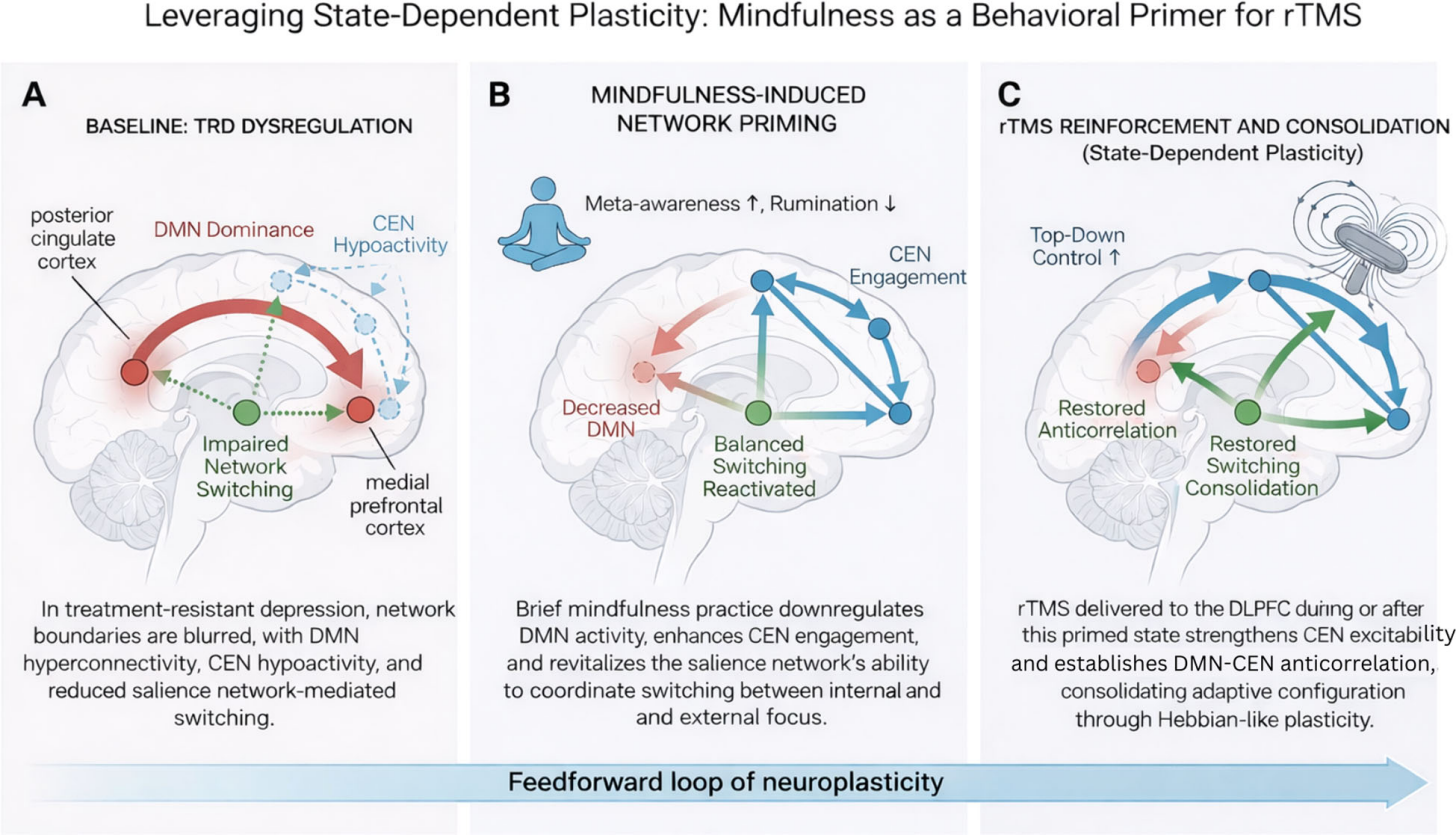
Leveraging state-dependent plasticity: Mindfulness as a behavioral primer for rTMS. **(A)** In treatment-resistant depression (TRD), hyperconnectivity within the Default Mode Network (DMN, red) and reduced Central Executive Network (CEN, blue) activity reflect impaired Salience Network (SN, green) switching and blurred network boundaries (16). **(B)** Brief mindfulness practice downregulates DMN dominance, enhances CEN engagement, and reactivates SN-mediated flexibility—priming the brain for efficient neuromodulation. **(C)** rTMS delivered to the dlPFC during or after this primed state strengthens CEN excitability and re-establishes DMN–CEN anticorrelation through state-dependent potentiation of targeted executive circuits (5). The combined approach integrates endogenous state modulation with exogenous stimulation, restoring large-scale network balance. Cumulative Synergy, The horizontal feedforward arrow illustrates a cycle of neuroplasticity; repeated sessions consolidate these functional shifts into stable trait-level changes, restoring large-scale network balance and structural-functional integrity.

The therapeutic efficacy of MBIs is increasingly understood through the framework of the Triple Network Model, which encompasses the Default Mode Network (DMN; primarily anchored in the medial prefrontal cortex [mPFC] and posterior cingulate cortex [PCC]), the Central Executive Network (CEN; comprising the dorsolateral prefrontal cortex [dlPFC] and posterior parietal cortex [PPC]), and the Salience Network (SN; characterized by the anterior insula [AI] and dorsal anterior cingulate cortex [dACC]). The Triple Network Model provides a framework for organizing neural activity into these three systems (2). While the CEN is thought to dominate under task-positive externally oriented conditions, the DMN is often characterized as its task-negative internally oriented counterpart (**Figure 1A**). Within this model, the SN is posited to evaluate salient internal and external stimuli and facilitate the dynamic switching between these two networks. In depression, this dynamic balance is disrupted: the DMN becomes hyperconnected and self-referential, the CEN is hypoactive, and the SN fails to efficiently regulate transitions between the two (2, 5–7).

MBIs have been shown to target the very circuits implicated in TRD pathogenesis. For instance, recent evidence suggests that mindfulness-based techniques can significantly reduce hyperconnectivity within the DMN (8, 9), potentially lowering the baseline of self-referential rumination. Specifically, comparing brain activation during mindfulness meditation versus a resting state reveals decreased activity in DMN subsystems. This finding is further clarified by Garrison et al. (10), who demonstrated that meditators show reduced DMN activity—specifically within the PCC and ACC—not only compared to a resting baseline but also compared to other active cognitive tasks (10). This suggests that mindfulness meditation is uniquely effective at suppressing self-referential thinking and mind-wandering, more so than simply occupying the mind with an effortful task. The authors interpret this as a diminished involvement in the habitual mode of self-reference.

However, the impact of mindfulness on the DMN is characterized by complex reorganization rather than global deactivation. While active meditation may quiet certain DMN nodes, meditators have been shown to exhibit greater resting-state functional connectivity within the DMN compared to nonmeditators (11). Furthermore, experienced practitioners demonstrate increased functional connectivity between the PCC (a DMN hub) and regulatory hubs of the SN (dACC) and CEN (dlPFC) during both rest and meditation (12). These findings suggest that mindfulness training facilitates increased conflict monitoring and heightened cognitive control over DMN functions, providing a robust neural substrate for enhanced self-regulation (4, 12). Crucially, these interventions may address the switching failures seen in TRD by modulating SN-mediated transitions; recent findings indicate that mindfulness training can shift brain dynamics during depressive rumination, allowing for more fluid and adaptive movement between internal and external attention streams (13).

Repetitive transcranial magnetic stimulation (rTMS) serves as a potent exogenous tool for modulating neuronal excitability and inducing neuroplasticity (6). While the primary stimulation site for rTMS in the treatment of TRD is typically the left dlPFC, its therapeutic efficacy is increasingly understood as a circuit-based phenomenon. The dlPFC serves as a critical entry point to the CEN, which is frequently found to be hypoactive in depressed states (6). HF-rTMS acts to increase neuronal excitability at this site, theoretically up-regulating the CEN to improve cognitive control and emotional regulation (5). However, the most compelling evidence for rTMS’s mechanism of action lies in its transsynaptic effects on distal, deeper brain structures. HF-rTMS of the dlPFC is thought to down-regulate overactivity in the subgenual anterior cingulate cortex (sgACC), a deep brain structure crucial for regulating negative affect through connectivity with DMN and SN (5, 6). Indeed, direct modulation of the sgACC with deep brain stimulation is an effective treatment for depression through modulation of DMN and SN activity (14). However, the therapeutic efficacy of rTMS appears to be highly state-dependent, potentially contingent upon the baseline integrity of the patient’s neural networks. Specifically, baseline connectivity within the DMN has emerged as a critical predictor of clinical response to TMS for TRD. This responder profile is characterized by robust internal communication within the DMN and, crucially, the preservation of clear functional boundaries—potentially expressed as strong anticorrelation—between the self-referential DMN and the task-oriented CEN (5, 7, 15, 16).

Recent precision mapping by Lynch et al. (16) has provided a rationale for these functional boundary failures in depression, demonstrating that the SN undergoes a pathological, nearly twofold expansion in many individuals with depression (16). This expansion allows the SN to effectively functionally encroach upon cortical territory typically occupied by the CEN and DMN, limiting the influence of executive and default mode networks. Such reorganization likely contributes to the frontostriatal and DMN dysfunctions identified by Liston et al. (5) and Drysdale et al. (15) that differentiate TMS responders from non-responders (5, 15). Further supporting this model, Zheng et al. (7) demonstrated in a large-scale cohort that the directional effective connectivity within these circuits—specifically within the DMN from mPFC to PCC—serves as a robust functional biomarker for predicting antidepressant treatment response, reinforcing the need for interventions that can acutely shift these network configurations (7). In summary, these findings highlight that the brain’s receptivity to TMS may be determined by its underlying network health—a state where both the physical organization of neural circuits and the quality of their functional communication dictate how effectively the stimulation can induce clinical recovery.

### A mechanistic framework for potential synergy between TMS and mindfulness-based interventions

Both TMS and MBIs engage large-scale neural networks implicated in self-regulation and mood, but they do so through complementary mechanisms. TMS exerts an exogenous modulation, altering neuronal excitability and synaptic plasticity via electromagnetic induction. In contrast, mindfulness functions as an endogenous modulation, training intrinsic attentional and emotional control systems through repeated mental practice. When considered together, these approaches converge on the Triple Network Model—modulating the DMN, CEN, and SN—suggesting that their combined or sequential application could produce synergistic network reorganization in TRD.

Liston et al. (5) identified that baseline hyperconnectivity between the sgACC, DMN, and CEN was a significant predictor of antidepressant TMS response (5). This suggests that the presence of functional crosstalk between these regions is a prerequisite for successful neuromodulation. Liston’s observation that a “primed” or highly connected circuit predicts better outcomes suggests that rTMS is most effective when the structural pathways for network-switching are intact. However, in many TRD patients, the SN’s capacity to dynamically toggle between these states may be impaired. MBI is proposed to induce a functional reorganization of the Triple Network by optimizing the dACC–PCC interface. By strengthening the connection between this SN hub and the DMN’s posterior node, mindfulness may enhance the brain’s endogenous capacity to regulate internal narrative streams, thereby preparing the circuit for more effective exogenous modulation via rTMS.

Indeed, successful TMS response is associated with restored anticorrelation between the CEN and DMN and improved integrity of salience-mediated switching mechanisms (5, 15). Mindfulness training may address these mechanisms by specifically altering brain dynamics during periods of depressive rumination, facilitating a more flexible transition between network states (13). Thus, both interventions act upon the same dysfunctional circuits, but from opposite directions—TMS may act to externally rebalance the system, while mindfulness could internally train the same networks to maintain that balance. Their overlap implies that mindfulness could serve as a neural primer, preparing the brain’s attentional and salience systems for more efficient TMS-induced modulation (**Figure 1B**). Characterizing these network shifts alongside changes in psychological domains may provide a more holistic view of clinical recovery. For example, recent observational work on other neuroplasticity-inducing interventions in TRD has highlighted the significant role of reducing cognitive and emotional rigidity while improving mentalization as indicators of treatment response (17). In the context of the MBI-rTMS synergy, these psychological markers could serve as clinical proxies for the functional decoupling of the DMN and CEN.

A growing body of evidence indicates that the plastic effects of TMS are highly state-dependent: when stimulation coincides with circuits already engaged in adaptive activity, it is more likely to induce Hebbian-like, long-term potentiation (LTP)-consistent changes—reinforcing those active pathways rather than perturbing them (18–20). High variability in rTMS outcomes may be largely due to its open-loop nature, which fails to account for the dynamic state of the target neural circuits (21). Without a method to monitor or target the brain’s immediate functional state, stimulation may be delivered during periods of high DMN activity or low CEN receptivity, potentially limiting the induction of neuroplasticity in these depression circuits. In TRD, the hyperconnectivity of the DMN and the hypoactivity of the CEN likely create a suboptimal baseline for plasticity.

While precision neuromodulation increasingly seeks technological solutions for adjusting treatment to real-time feedback, MBI offers a practical, endogenous method for closing the loop. This framework parallels established precision TMS paradigms, such as the Stanford Accelerated Intelligent Neuromodulation Therapy (SAINT) protocol and EEG-triggered closed-loop stimulation, which utilize individualized functional connectivity or real-time biomarkers to optimize stimulation timing and location. By framing mindfulness as a behavioral endogenous parallel to these technological approaches, the proposed synergy model aligns with the broader move toward state-dependent, personalized neuromodulation.

By identifying and disengaging from maladaptive ruminative states in real-time, mindfulness practice may help ensure that the neuromodulatory pulse is delivered to a brain that is actively working to reorganize its own large-scale networks, thereby maximizing the therapeutic signal-to-noise ratio. In this framework, TMS and MBI operate as partners in a feedforward loop of neuroplasticity (**Figure 1C**). A brief mindfulness session before stimulation aligns the patient’s large-scale networks toward a more coherent, anticorrelated state (DMN downregulation, CEN upregulation, SN synchronization). TMS then reinforces this configuration through exogenous excitation of the same nodes, strengthening synaptic connections consistent with the ongoing mental state. Post-stimulation mindfulness practice could further consolidate these changes, stabilizing the newly potentiated network architecture through continued self-regulatory engagement.

Beyond the neurobiological advantages, this multimodal approach may significantly enhance patient agency. For individuals with TRD—who often feel disempowered by a history of failed treatments—the active role required by MBI changes the treatment narrative from one of passive reception to one of active mastery. This increased self-efficacy may independently contribute to long-term resilience and a reduction in post-treatment relapse rates.

## Results

### Evidence from analogous combined therapies

The current literature of combined rTMS and behavioral interventions is summarized in **Table 1**, which distinguishes between direct rTMS-mindfulness trials and analogous state-engaged behavioral protocols.

**Table 1:** Summary of clinical and analogous studies combining rTMS with behavioral state-manipulation in depression.

Section A: Direct evidence for rTMS + mindfulness/meditation synergy						
Study/author (year)	Population	rTMS protocol (type; target; sessions)	MBI modality & dose	Timing	Primary outcomes & follow-up	Key notes/feasibility
Pradhan et al. (25)	TRD (N = 1 Case Report)	10 Hz rTMS; Target: dlPFC; Sessions: 30 sessions (5 times/week over 6 weeks).	MBCT + Focused Attention Meditation; Dose: 20 sessions (5 min pre-TMS, 30 min during TMS, and 10–15 min MBCT at the end of TMS).	During (Concurrent)	Remission achieved (HAM-D17 dropped from 32 to 6). Sustained remission at 8-month follow-up.	Utilized real-time EEG neurofeedback (MUSE headband). Patient achieved remission with fewer TMS sessions than standard (30 vs. 36) and tapered off 2 of 3 medications.
Cavallero et al. (26)	MDD	10 Hz (or 1 Hz right/20 Hz left); Target: Left dlPFC (or bilateral); Sessions: Average 36 sessions (5 weeks).	Audio-guided MBCT; Dose: 5 to 39 min/day.	During (Simultaneous)	Significant reductions in IDS-SR and PHQ-9 post-treatment. Poor feasibility: high dropout rate (10 of 27 withdrew).	Feasibility was hindered by TMS tapping noise and physical sensations, which disrupted focus and induced anxiety/negative mood states in some patients.
Duan et al. (27)	Post-Stroke Depression (PSD)	10 Hz rTMS; Target: Left dlPFC; Sessions: 20 sessions over 4 weeks.	MBSR; Dose: 6 weekly 1-hour sessions + 20 min/day homework.	During (Audio played throughout TMS) & Independent sessions	Improvements in HAMD-17, MMSE, MBI, and PSQI post-treatment and sustained at 8-week follow-up.	N=71 RCT. rTMS+MBSR was highly feasible with no reported headaches. Cognitive function (MMSE) at baseline correlated with depression prognosis.
MEND Trial (Ongoing)	MDD	iTBS; Target: Left dlPFC; Sessions: 20 treatments over 4 weeks.	Digital mindfulness training (focused on breathing); Dose: Evaluating 10-min vs. 20-min session doses.	Concurrent	Primary outcome: Change in EEG source-localized pDMN alpha activity at 4 weeks. Secondary: MADRS/PHQ9 at 8 weeks.	Phase 2 trial. Determines optimal dose for neural target engagement relative to an active control (intermittent deep breathing + iTBS).

Section B: Analogous behavioral state-manipulation + rTMS studies						
Study/author (year)	Population	rTMS protocol (type; target; sessions)	Behavioral/cognitive task & dose	Timing	Primary outcomes & follow-up	Key notes/feasibility
Donse et al. (24)	MDD & Dysthymia (>97% treatment-resistant to 1+ antidepressant)	10 Hz or 1 Hz; Target: Left dlPFC (10 Hz) or Right dlPFC (1 Hz); Sessions: Average 20.9 sessions; minimum frequency of 2 to 3 times per week, and a maximum frequency of 2 times per day	CBT-based Psychotherapy; Dose: 45-minute sessions.	Concurrent + post-TMS: for the first 20 minutes, the patient received rTMS while the therapist actively conducted psychotherapy. Once the magnetic pulses stopped, the psychotherapy continued for the remaining 25 minutes of the session	66.3% response, 56.0% remission at endpoint. Sustained response/remission seen at 6-month follow-up.	Large naturalistic cohort (N = 196). Early symptom change (BDI reduction at session 10) was highly predictive of overall treatment response.
Russo et al. (22)	TRD	Standard rTMS; Target: Left prefrontal cortex (implied); Sessions: Average 33 sessions over 6 weeks.	Behavioral Activation (BA); Dose: 5–10 min interval before sessions	Before TMS sessions	77% goal completion rate. Significant decreases in IDS-SR (47%), PHQ-9 (55%), and anhedonia (SHAPS).	Pilot study (N = 11). Easily incorporated by TMS technicians without disrupting clinical workflow; standardized the daily clinical assessments.
Fryml et al. (23)	PTSD in veterans	10 Hz rTMS (120% MT, 3000 pulses/session); Target: Left or Right prefrontal cortex; Sessions: 5 sessions (once weekly for 5 weeks).	Prolonged Exposure (PE) therapy (audio recording of imaginal exposure); Dose: ≥40 minutes (5 min pre-TMS, 30 min during TMS, ≥5 min post-TMS).	5 min pre-TMS, 30 min during TMS, ≥5 min post-TMS	Nonsignificant trend toward PTSD improvement (CAPS scores); significant antidepressant benefit for comorbid MDD.	N=12 (8 completed. Demonstrated safety and feasibility. The 34% dropout rate was roughly equivalent to standard PE therapy, suggesting the addition of rTMS was well tolerated.

Although direct evidence for pairing TMS with meditation remains limited, analogous combination therapies demonstrate that pairing TMS with concurrent psychological or behavioral interventions can amplify antidepressant response by leveraging the neuroplasticity induced by stimulation (22–24). Early, underpowered pilot studies of combined neuromodulation have yielded promising but inconclusive results (22, 23). Larger-scale data suggest that the integration of rTMS with behavioral interventions may outperform monotherapy (24). In a large cohort of patients with major depressive disorder (MDD) (n=196), Donse et al. (24) demonstrated that the concurrent dlPFC rTMS with psychotherapy resulted in a 66% response rate and a 56% remission rate—outcomes that may be better than often observed for rTMS alone, and suggesting that the cognitive engagement of a circuit may be critical for translating neuroplastic changes into clinical recovery (24).

While studies such as Donse et al. (24) demonstrate high remission rates for combined rTMS and psychotherapy, it is important to note that these results were derived from broader MDD populations. While the clinical history of the participants suggests a high likelihood of treatment resistance, the study did not utilize formal TRD inclusion criteria or stratify outcomes based on previous treatment failures. Consequently, applying these findings to a strictly defined TRD population requires careful extrapolation, as the neurobiological heterogeneity and extensive treatment history of TRD patients may influence their receptivity.

Beyond circuit-level priming, it is important to acknowledge alternative explanations for the enhanced outcomes observed in these combined therapies. Factors such as the simple additive effect of two independent antidepressant interventions, an enhanced therapeutic alliance resulting from increased clinical contact, or non-specific behavioral activation may contribute significantly to clinical recovery.

While this analogous treatment combination highlights the promise of leveraging state-dependent neuroplasticity, direct clinical research pairing TMS with mindfulness remains in its early stages. A case-study by Pradhan et al. (25) described remission in a patient with TRD treated with concurrent TMS and focused-attention meditation, offering an important proof of concept (25). A later pilot study by Cavellero et al. (26) had participants engage in audio-guided meditation during TMS sessions but found the percussive sound and somatosensory sensations of the TMS equipment to be ultimately too distracting from the guided meditations (26). More robust evidence for TMS-MBI synergy emerged later in the form of a randomized controlled trial of 71 patients: Duan et al. (27) demonstrated that the combination of rTMS applied over the left dlPFC and Mindfulness-Based Stress Reduction (MBSR) was significantly more effective than MBSR combined with sham rTMS or sham rTMS combined with general psychological care (27). Patients receiving the combined treatment showed superior improvements in depressive symptoms (HAMD/SDS scores), cognitive function (MoCA), and activities of daily living. This clinical success underscores the potential for mindfulness to synergistically enhance neuromodulation, while the practical challenges noted by Cavellero et al. (26) highlight the importance of refined, temporally sequenced protocols—such as meditation immediately before or after stimulation—to maximize circuit engagement without the sensory interference of active meditation instruction. Ongoing investigations include the Mindfulness Engaged Neurostimulation for Depression (MEND) trial (28). This clinical trial utilizes iTBS combined with digital mindfulness training to investigate its efficacy in suppressing posterior Default Mode Network (pDMN) brain activity, as measured by electroencephalography (EEG). By evaluating the optimal dose required for neural target engagement relative to an active control training, the MEND trial aims to determine if this multimodal approach can successfully enhance cognition and alleviate depression symptoms in patients with TRD.

## Discussion

### Methodological challenges and future directions

A significant challenge in the study of MBIs is the heterogeneity of practices and delivery methods, which complicates standardization and reproducibility. MBIs encompass a wide range of techniques—from formal sitting meditation and body scans to informal practices such as mindful walking or breath awareness—and studies have employed diverse delivery formats. For instance, some investigations have relied on pre-recorded digital audio guidance (25, 28), whereas others have used live instruction from certified mindfulness teachers (27). This variability represents a key limitation, as both the dose and context of practice likely influence the desired changes to the DMN, CEN, and SN.

A second methodological gap concerns the temporal alignment of mindfulness practice with stimulation. The principle of context-dependency in TMS is well established: the physiological and behavioral effects of stimulation vary according to the functional activation state of the targeted neural population at the time of the TMS pulse (18). More recent work has emphasized the potential importance of the brain’s functional state in clinical rTMS protocols, noting that conventional approaches typically assume a resting state but may miss opportunities to leverage state-dependent responsiveness for improved efficacy (20). Building on the state-dependent framework, it is reasonable to hypothesize that inducing a meditative state immediately before stimulation—characterized by down-regulated DMN activity and enhanced prefrontal control—could influence the ensuing neuroplastic response. This possibility remains untested but offers a clear and mechanistically grounded avenue for future investigation. Most existing studies have implemented mindfulness during or immediately after stimulation; future research should therefore examine the temporal dynamics of this pairing to determine whether pre-stimulation mindfulness induction can optimize network states for TMS responsiveness. Rather than a simple additive effect, this approach may represent a deliberate, synergistic interaction in which mindfulness modulates *state* and rTMS modifies *structure*, each amplifying the other’s impact.

Future trials should also incorporate transdiagnostic mechanistic endpoints that capture the restoration of higher-order cognitive capacities. Adopting validated tools to assess metacognition—the ability to monitor and integrate mental states—could offer a more granular understanding of how behavioral priming facilitates durable circuit-level reorganization (29). Tracking these capacities alongside connectivity metrics would allow for a more balanced appraisal of the effects proposed in this framework.

Future research should therefore prioritize (1) comparing different mindfulness modalities (focused attention vs. open monitoring), (2) defining the optimal temporal sequence of mindfulness relative to TMS pulses, (3) integrating fMRI and electrophysiological measures to map real-time changes in DMN and salience network activity, while also utilizing repeated Diffusion Tensor Imaging (DTI) and resting-state fMRI (rs-fMRI) to measure longitudinal structural and functional plasticity, and 4) incorporating mechanistic psychological endpoints—such as metacognitive capacity, mentalization, and cognitive-emotional rigidity—which may serve as measurable clinical proxies for circuit integrity. Standardized, multimodal protocols tested in adequately powered randomized trials will be crucial for determining the clinical significance of this proposed synergy. While meta-analytic evidence increasingly confirms the capacity of MBI to reorganize large-scale brain networks (9), the field still requires large-scale, independent replication studies specifically within clinical TRD populations to define optimal dosing and timing.

### Testable predictions of the synergy model

The synergy model leads to several testable predictions:

1. Pre-rTMS mindfulness will significantly reduce DMN dominance (state markers) and increase CEN receptivity to TMS compared to sham mindfulness.
2. The magnitude of these pre-session DMN>CEN shifts will positively correlate with longitudinal depressive symptom improvement.
3. Synergistic antidepressive effects of TMS-MBI will be most pronounced in patients exhibiting high baseline rumination and elevated DMN-sgACC coupling.
4. The combined rTMS + MBI intervention will demonstrate a statistically significant interaction effect.

To facilitate clinical translation, we recommend a 2×2 factorial RCT design consisting of four distinct arms: (1) Active rTMS + Active MBI; (2) Active rTMS + Sham MBI; (3) Sham rTMS + Active MBI; and (4) Sham rTMS + Sham MBI. Synergy would be statistically defined by a significant interaction term, demonstrating that the combined clinical effect exceeds the cumulative benefits of mindfulness and rTMS administered in isolation. Mechanistic endpoints should include rs-fMRI connectivity, EEG biomarkers, and validated rumination scales. Clinically, a feasibility-focused workflow might involve 10 minutes of guided mindfulness in a quiet room immediately preceding coil placement to avoid the sensory distraction inherent in simultaneous delivery.

## Conclusion

While the field of precision psychiatry continues to develop sophisticated external monitoring systems—using real-time EEG or fMRI to capture dynamic brain states and distribute stimulation accordingly (19, 21)—this review argues that MBI may offer a parallel, endogenous solution. Mindfulness practice trains the patient to recruit and develop their own internal monitoring system. By strengthening the functional connectivity between the SN and the DMN (9), mindfulness may enhance meta-awareness. This can allow a patient with depression to actively downregulate ruminative noise and engage the CEN in real-time, essentially self-inducing a better baseline state for neuroplasticity and clinical response to rTMS.

In this model, the patient is not simply a passive recipient of a pre-programmed pulse, but an active collaborator who “closes the loop” from the inside out. By aligning the extrinsic neuromodulatory force of rTMS with the intrinsic regulatory skill of mindfulness, clinicians can target the triple network dysfunction of TRD from both angles. Further research is needed to operationally define and empirically test whether these interventions yield true synergistic interactions rather than purely additive benefits.

While early case and pilot studies provide encouraging signals, the field still lacks standardized protocols and clear guidance on timing. Large-scale controlled trials, such as the ongoing MEND study, will be essential to validate these mechanisms and define the clinical significance of pre-stimulation priming. Ultimately, integrating mindfulness with TMS offers a pathway toward multimodal psychiatry that harnesses both the brain’s biological plasticity and the mind’s capacity for self-regulation to achieve more durable remission.
